# Quantification of upper limb position sense using an exoskeleton and a virtual reality display

**DOI:** 10.1186/s12984-018-0367-x

**Published:** 2018-03-16

**Authors:** Anne Deblock-Bellamy, Charles Sebiyo Batcho, Catherine Mercier, Andreanne K. Blanchette

**Affiliations:** 1 Center for Interdisciplinary Research in Rehabilitation and Social Integration (CIRRIS), 525 Boulevard Wilfrid-Hamel, Quebec City (QC), G1M 2S8 Canada; 2 Faculty of Medicine, Universite Laval, 1050 Avenue de la Medecine, Quebec City (QC), G1V 0A6 Canada; 3 Department of Rehabilitation, Universite Laval, 1050 Avenue de la Medecine, Quebec City (QC), G1V 0A6 Canada

**Keywords:** Assessment, Proprioception, Robotic, Stroke, Sensory, Virtual reality, Upper limb

## Abstract

**Background:**

Proprioceptive sense plays a significant role in the generation and correction of skilled movements and, consequently, in most activities of daily living. We developed a new proprioception assessment protocol that enables the quantification of elbow position sense without using the opposite arm, involving active movement of the evaluated limb or relying on working memory. The aims of this descriptive study were to validate this assessment protocol by quantifying the elbow position sense of healthy adults, before using it in individuals who sustained a stroke, and to investigate its test-retest reliability.

**Methods:**

Elbow joint position sense was quantified using a robotic device and a virtual reality system. Two assessments were performed, by the same evaluator, with a one-week interval. While the participant’s arms and hands were occluded from vision, the exoskeleton passively moved the dominant arm from an initial to a target position. Then, a virtual arm representation was projected on a screen placed over the participant’s arm. This virtual representation and the real arm were not perfectly superimposed, however. Participants had to indicate verbally the relative position of their arm (more flexed or more extended; two-alternative forced choice paradigm) compared to the virtual representation. Each participant completed a total of 136 trials, distributed in three phases. The angular differences between the participant’s arm and the virtual representation ranged from 1° to 27° and changed pseudo-randomly across trials. No feedback about results was provided to the participants during the task. A discrimination threshold was statistically extracted from a sigmoid curve fit representing the relationship between the angular difference and the percentage of successful trials. Test-retest reliability was evaluated with 3 different complementary approaches, i.e. a Bland-Altman analysis, an intraclass correlation coefficient (ICC) and a standard error of measurement (SEm).

**Results:**

Thirty participants (24.6 years old; 17 males, 25 right-handed) completed both assessments. The mean discrimination thresholds were 7.0 ± 2.4 (mean ± standard deviation) and 5.9 ± 2.1 degrees for the first and the second assessment session, respectively. This small difference between assessments was significant (− 1.1 ± 2.2 degrees), however. The assessment protocol was characterized by a fair to good test-retest reliability (ICC = 0.47).

**Conclusion:**

This study demonstrated the potential of this assessment protocol to objectively quantify elbow position sense in healthy individuals. Futures studies will validate this protocol in older adults and in individuals who sustained a stroke.

## Background

Proprioception is defined as the ability to perceive body segment positions and movements in space [1]. Sensory receptors involved in proprioception are mostly located in muscle [2–4], joint [5, 6] and skin [3, 7]. Proprioceptive sense is known to play a significant role in motor control [8–11] and learning [8, 12], particularly in the absence of vision. The importance of proprioceptive inputs has been demonstrated while studying individuals who presented lack of proprioception due to large-fiber sensory neuropathy [11, 12]. Despite an intact motor system, somatosensory deafferentation may lead to limitations in several activities involving motor skills, such as eating or dressing [12]. These disabilities may also be observed in individuals with proprioceptive impairments due to a stroke. Indeed, approximately half of the individuals who sustained a stroke present proprioceptive impairments in contralesional upper limb [13]. After a stroke, proprioception is known to be related to recovery of functional mobility and independence in activities of daily living (ADL; [14]). Fewer individuals with significant proprioceptive and motor losses (25%) were independent in ADL than individuals with motor deficits alone (78%). Moreover, fewer individuals with proprioceptive deficits (60%) after a stroke are discharged from the hospital directly to home compared to those without proprioceptive deficits (92%) [15].

Although the negative impact of proprioceptive impairments on motor and functional recovery is known, a large proportion of clinicians (70%) report not using standardised assessment to evaluate somatosensory deficits in patients with a stroke [16]. In clinical and research settings, proprioception is most frequently assessed with limb-matching tasks. Two types of matching tasks have commonly been used: the ipsilateral remembered matching task and the contralateral concurrent matching task [17]. In an ipsilateral remembered matching task, the evaluator or robotic device brings the patient’s limb to a target joint position, when the patient’s eyes are closed, keeps the limb in this position for several seconds, and then moves back the limb to the initial position. The patient needs to memorize the reference position and replicate it with the same (ipsilateral) limb. This task cannot, however, be used to evaluate proprioception in individuals with working memory issues, which represent around 25% of individuals who sustained a stroke [18]. In such cases, the matching error observed could reflect memory deficits, rather than proprioceptive impairments. Moreover, upper limb paresis affects 76% of individuals who sustained a stroke [19], making the task’s execution difficult or impossible. Assessing proprioception with the less affected arm as the indicator arm is therefore frequently considered in patients with hemiparesis. Indeed, in a contralateral concurrent matching task, the patient has to reproduce a mirror image of the evaluated limb position with the opposite (contralateral) limb [17]. However, considering that 20% of individuals who sustained a stroke also presents proprioceptive impairment on the ipsilateral side of the lesion [13], it would be difficult to ascertain whether the error is due to deficits in the evaluated arm, the opposite arm or both. In addition, interhemispheric communication is required in a contralateral concurrent matching task. Individuals with asymmetric stroke or with transcallosal degeneration would therefore be particularly disadvantaged while being assessed with a contralateral concurrent matching task [17].

In order to study proprioception in individuals who sustained a stroke, we developed an assessment protocol, that combines the use of an exoskeleton and a virtual reality system, enabling the quantification of position sense without using the opposite arm, involving active movement of the evaluated limb or relying on working memory. The primary objective of the present study was to validate the assessment protocol by quantifying the elbow joint position sense of healthy adults, before using this protocol with individuals who sustained a stroke. As a secondary objective, test-retest reliability of the assessment protocol was investigated.

## Methods

### Participants

Participants were recruited in this descriptive study using a convenience sample. They were included if they were between 18 and 55 years old and were excluded if they had any history of musculoskeletal disorder of the dominant upper limb or any neurological condition that could affect their performance. This study was approved by the institutional ethics review board. All subjects provided written informed consent prior to their participation in the study. Based on sample size formula proposed by Zou [20], it was determined that a minimal sample size of 19 participants was required for this experiment (hypothesized intraclass correlation coefficient = 0.7; α=0.05; β=0.95). According to Springate [21], a sample of at least 25–30 participants should be used to provide an adequate estimate of the random error. A sample size of 30 participants was therefore recruited to evaluate the stability of the assessment protocol.

### General procedure

Participants came to the Center for Interdisciplinary Research in Rehabilitation and Social Integration (Quebec City, Canada) for two assessment sessions with a one-week interval between sessions. At the beginning of the first session, each participant read and signed the consent form and completed sociodemographic and general health questionnaires. These questionnaires were used to determine participants’ eligibility (history of neurological and/or musculoskeletal disorders), to establish their sociodemographic profile (age, gender, dominance, weight, height, occupation, and leisure activities) and to compare qualitatively their level of perceived fatigue and stress at the beginning of both assessment sessions. After the participant’s installation, the assessment of dominant upper limb proprioception was performed. The duration of this first session was approximately 90 min, including 40–45 min for the proprioception assessment itself. The second session was devoted exclusively to the proprioception assessment and the total duration was approximately 45 min. Four different evaluators participated in this experiment. For each participant, the same evaluator carried out both assessments.

### Proprioception assessment

The assessment protocol enabled the identification of the smallest angular difference, between the participant’s arm and a virtual arm representation projected on a screen located over participant’s arm, that each participant was able to discriminate (discrimination threshold) using a two-alternative forced choice paradigm.

**Fig. 1:**
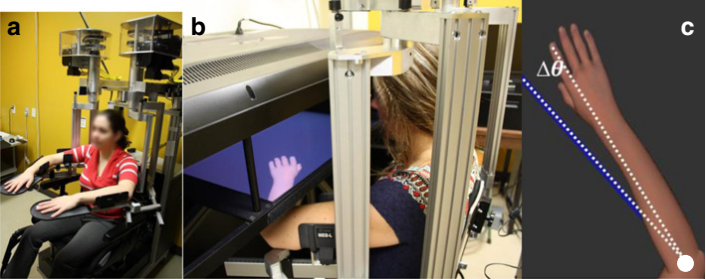
KINARM Exoskeleton Lab. **a** Modified wheelchair with each arm supported against gravity by exoskeletons; (**b**) Virtual reality display; (**c**) Virtual arm and real arm positions (blue line; non-visible for the participant) where ∆Θ represents the angular difference between the real and the virtual arm. The white circle corresponds to the center of rotation, i.e. the elbow joint

The proprioception assessment was performed using the KINARM Exoskeleton Lab (BKIN, Technologies Ltd., Kingston, Ontario, Canada). It consists of 2 motorised upper limb exoskeletons (Fig. 1a) that enable passive arm movements in the horizontal plane and a two-dimensional virtual reality (VR) display. This VR system projected a virtual arm representation on a screen placed over the participant’s upper limb (Fig. 1b). The KINARM Exoskeleton Lab has been previously used to assess proprioception and motor functions in stroke [22–26] and traumatic brain injury adult populations [27]. However, in these studies, a contralateral concurrent matching task was used to assess position sense [22–26], contrary to the present experiment.

During the proprioception assessment, participant sat in a wheelchair base with each arm supported against gravity by the exoskeletons (wrist in neutral position, forearm in pronation, shoulder at 65 degrees of horizontal abduction). At first, the evaluator adjusted the exoskeletons to fit in the participant. These adjustments were recorded and reproduced in the second session. The exoskeleton and VR display were then calibrated, in accordance with the company recommendations, to correctly measure arm position data and to properly align the virtual representation. The alignment and the length of the virtual forearm were determined based on the participant’s arm. The size of the hand was proportionally adjusted based on the forearm dimensions.

**Fig. 2:**
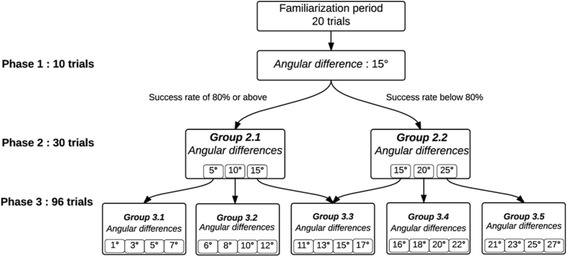
Schematic representation of the decision tree used in the experimental protocol. If the participant was able to successfully identify 80% of trials or more in the first phase, the angular differences became smaller for the second phase (5, 10, 15°). If not, the angular differences used in the second phase were larger (15, 20, 25°). The second phase included 30 trials. The success rate reached in the second phase determined the selection of the angular differences tested in the third phase. For instance, if a participant did not reach at least 80% of correct identification of position with a 10-degree difference, but successfully detected a 15-degree difference in ≥80% of the trials, the angular differences tested in the next phase would be 11, 13, 15, and 17°. Within each phase, angular differences changed pseudo-randomly

In each trial, the exoskeleton passively moved the dominant forearm (towards elbow flexion or extension) from an initial position to a target position. Shoulder position was maintained at 65° of horizontal abduction throughout the entire protocol (0 degrees of horizontal abduction corresponding to arm raised to the side). The upper limbs were occluded from vision. Four different initial positions (30, 55, 70, 95° of elbow flexion) and two target positions (50 and 75° of elbow flexion) were used and randomized to avoid anticipation from the participants. The amplitude of the angular displacement between initial and target position was systematically 20° and the displacement was performed in 4 s, corresponding to a velocity of 5 deg./s, for each trial. Elbow joint angular positions and velocities were obtained from KINARM motor encoders. Once the robot completed the passive displacement towards target position, a virtual arm representation was projected on the screen. This virtual representation was not perfectly superimposed over the real arm, however. The angular difference (Fig. 1c) between the participant’s arm and the virtual representation ranged from 1° to 27°, towards flexion or extension and changed pseudo-randomly across trials. For each trial, the participant was instructed to verbally identify the relative position of their arm (more flexed or more extended) compared to the virtual representation. Each assessment consisted of 136 trials, distributed in three phases of 10, 30 and 96 trials (Fig. 2). Verbal answers given by the participant were noted by the evaluator. No feedback about results was provided to the participant during the task.

The assessment began with a period of familiarization, including a total of 20 trials. Indeed, 10 trials were first done with a visual feedback of the real arm to confirm the participant’s understanding of the instructions. Then, the participant had to complete 10 additional trials without visual feedback, to familiarize himself with the virtual environment and the experimental task. During this familiarization phase (not in the other phases), a total of 4 trials were performed when the virtual representation was exactly superimposed over the real arm in order to verify KINARM’s calibration. Considering that the protocol aimed at identifying a precise discrimination threshold in as few trials as possible for each participant, the difficulty level of the experimental task was adapted to the participant’s capacity based on a decision tree (Fig. 2). The angular difference was initially set at 15° for the first phase (10 trials), and then adjusted in the following phases according to the participant’s percentage of successful trials obtained in the previous phase, based on an adaptive psychophysical method. As described in Taylor and Creelman [28], an adaptive method consists of a sequence of trials, which begins by testing at some arbitrary initial level (Phase 1). After a predefined number of trials, a new testing level is determined, and so on (Phases 2 and 3). In the second and third phases, several angular differences were tested repeatedly in random order. An angular difference successfully identified in 80% of the trials or more was considered as detected. Participants were therefore categorized based on their capacity to detect elbow joint position.

Preliminary data showing the feasibility of using this assessment protocol in individuals with a stroke have been published in abstract form [29].

### Statistical analysis

Parametric descriptive statistics, such as mean, standard deviation (SD) and frequency, were used to describe sociodemographic data (age, gender, dominance).

**Fig. 3:**
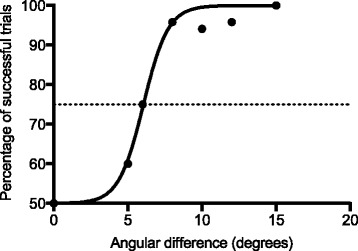
Sigmoid curve fit representing the relationship between the angular difference and the percentage of successful trials in a representative participant (Group 3.2). This figure combined results of Phase 1, 2 and 3

To quantify position sense for each participant, a discrimination threshold was statistically extracted from a sigmoid curve fit representing the relationship between the angular difference (x) and the percentage of successful trials (y) (Fig. 3). The best curve fit was found through the least squares (ordinary) fitting method. For each assessment, a discrimination threshold corresponding to the angular difference at 75% of successful trials was calculated based on the following 4-parameter logistic equation:

$$ y= Lower+\frac{\left( Upper- Lower\right)}{1+{10}^{\left( LogEC50-X\right)\ast HillSlope}} $$

Considering the two-alternative forced choice paradigm, Lower (Y value at the lower plateau) and Upper (Y value at the upper plateau) were fixed at 50 and 100%, respectively. EC50 corresponds to the X value when the Y is halfway between the Upper and Lower values. HillSlope describe the steepness of the curve. Similar methods of discrimination/detection threshold identification were used in previous studies [30–33].

In order to assess test-retest reliability, different complementary approaches were used, as recommended in Lexell et al. [34]. A Bland-Altman plot was used to reflect the spread of the score difference (Session 2 - Session 1) around the mean discrimination threshold and to determine the presence or absence of a bias between discrimination thresholds obtained in both assessments. Relative reliability, referring to the degree to which individuals maintain their position in a sample with repeated measurements [35], was quantified with a two-way mixed single measure ICC (2,1) for absolute agreement. ICC was interpreted according to Fleiss [36], where an ICC < 0.4 indicates a poor reproducibility, an ICC between 0.4 and 0.75 indicates a fair to good reproducibility and an ICC ≥ 0.75 indicates an excellent reproducibility. Absolute reliability, referring to the variability of the scores from trial to trial which is not dependent on the sample [37], was obtained with the standard error of measurement (SEm). The SEm, calculated as follows $\mathrm{SEm}=\sqrt{\mathrm{WMS}}$ (where WMS represents the mean squared error term extracted from the repeated measures analysis of variance [34]), is an indicator of the dispersion of the measurement errors to estimate participant’s scores from their observed score [38].

Data analyses and statistics were performed using Microsoft Excel 15.18 (Microsoft Corporation, Redmond, Washington, USA), GraphPad Prism 7 (GraphPad Software, Inc., La Jolla, California, USA), IBM SPSS Statistics 24.0 (SPSS Inc., Chicago, Illinois, USA) and R 3.3.3 (The R Foundation for Statstical Computing, Vienna, Austria).

## Results

Thirty healthy adults (17 males, 25 right-handed, mean age ± SD: 24.6 ± 3.3 years old) participated in the present study.

The distribution of participants across categories, representing their capacity to discriminate elbow joint position, can be observed in Table 1. In Phase 2, all participants, except one, were categorized in Group 2.1, regardless of the assessment session. In Phase 3 of the Session 1, participants were distributed across Groups 3.1, 3.2 and 3.3, with a majority (53.3%) in Group 3.2. In Session 2, these percentages were slightly different, with percentage of participants almost equally distributed between Groups 3.1 and 3.2. Modifications on the categorization across sessions were therefore observed. While 14 participants remained in the same group, 13 participants moved to a group testing smaller errors, and 3 participants moved to a group testing larger errors.

**Table 1:** Participants’ categorization based on their capacity to detect elbow joint position

Group	Session 1 n (%)	Session 2 n (%)
Phase 2		
2.1	28 (93.3%)	30 (100%)
2.2	2 (6.6%)	0 (0%)
Phase 3		
3.1	8 (26.7%)	14 (46.7%)
3.2	16 (53.3%)	15 (50.0%)
3.3	6 (2.0%)	1 (3.3%)

### Quantifying the elbow position sense of healthy participants

**Fig. 4:**
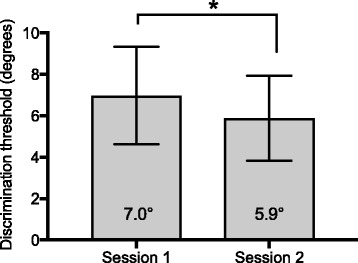
Mean discrimination threshold (degree) for each assessment session. Error bars represent SD. * = *p* < 0.05

Mean discrimination thresholds of healthy participants were 7.0° ± 2.4° and 5.9° ± 2.1° (mean ± SD) for Sessions 1 and 2, respectively (Fig. 4), with a mean difference between assessments of − 1.1° ± 2.2°.

In Session 2, participants included in Group 3.1 had discrimination thresholds ranging from 3.0 to 6.3 degrees, while discrimination thresholds measured in Group 3.2 participants ranged between 5.6 and 8.3 degrees. The only participant categorized in Group 3.3 had a discrimination threshold of 14.0 degrees.

Mean coefficients of determination (R-squared), indicating the goodness of the sigmoid curve fit, were 0.91 ± 0.07 (range: 0.77–1.0) for Session 1 and 0.93 ± 0.05 (range: 0.76–1.0) for Session 2.

### Test-retest reliability

**Fig. 5:**
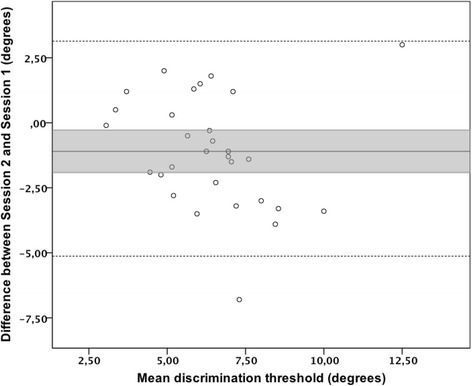
Bland-Altman plot of the differences (*n* = 30) between Session 2 and Session 1 vs. the mean of both assessments; black solid line = mean difference; dashed lines = limits of agreement; grey area = mean difference ± 95%CI

A Bland-Altman plot representing the differences between discrimination threshold measured in Sessions 2 and 1 as a function of the mean of both assessments showed that the line of equality (Mean difference = 0) is located outside the 95% confidence interval (95% CI) of the mean difference (Fig. 5), suggesting a significant bias [34] of − 1.1° ± 2.2°. The intraclass correlation coefficient (ICC) for absolute agreement between discrimination threshold measured in Sessions 1 and 2 was 0.47 (95% CI: 0.14–0.71) indicating a fair to good test-retest reliability. Moreover, the SEm was 1.5°, which represents 25.4% of the mean discrimination threshold measured in the second session.

## Discussion

We developed a position sense assessment protocol, which combines a robotic device and a virtual reality display, that enables the quantification of elbow position sense without using the opposite arm, involving active movement of the evaluated limb or relying on working memory, considering these abilities may be impaired after a stroke. As a preliminary step of validation, this experiment showed that this assessment protocol can quantify elbow position sense of healthy young adults (mean threshold: around 6.5 degrees), with a fair to good test-retest reliability. Moreover, it enabled quantification on a continuous scale, as demonstrated by the overlap in discrimination thresholds despite participants’ categorization (Group 3.1 = 3.0–6.3 degrees; Group 3.2 = 5.6–8.3 degrees in Session 2).

### Quantifying the elbow position sense

This new proprioception assessment protocol can objectively quantify the position sense with a discrimination threshold (in degrees), contrary to the most commonly used proprioception clinical assessments, which are predominantly using ordinal or dichotomous scales. The Rivermead Assessment of Somatosensory Performance (RASP) and the Nottingham Sensory Assessment (NSA) were identified, by clinicians, as the most frequently used standardised somatosensory assessments that include a proprioception testing [16]. The NSA evaluates somatosensory impairments through 7 sub-tests, including an appreciation of movement perception and direction. The patient is asked to reproduce a mirror image of the movement with the other arm, while he/she is blindfolded. Proprioception is then evaluated on a 4-level ordinal scale, where 0 = no movement perceived; 1 = appreciation of the movement taking place; 2 = direction of movement sense, but inaccurate position; 3 = joint position sense, within 10° of the position [39]. The RASP evaluates 7 different sensory modalities, including joint movement and movement direction discrimination. The patient has to report orally if a movement is perceived (movement discrimination) and in which direction the segment is moved (“up” or “down”; direction discrimination). For each trial, proprioception movement and direction discrimination are evaluated using a dichotomous scale, where 0 represents a missed trial, and 1 represents a successful trial [40]. These clinical tools present some limitations for proprioception assessment, however. The identification of proprioceptive deficits may be possible with these tools, but it remains difficult to assess the severity of impairment and demonstrate patient’s progression while using dichotomous or ordinal scales. Moreover, the evaluator has to subjectively evaluate the participant’s capacity. These tests cannot be used in healthy subjects (ceiling effect) and, consequently, standard norms cannot be determined to facilitate the interpretation of the score. Contrary to the RASP and the NSA, our proprioception assessment protocol allows a quantification of elbow position sense on a continuous scale. Consequently, it is reasonable to believe that it enables a more precise measurement and provides a more sensitive assessment, which is essential to evaluate the effectiveness of a clinical intervention.

In an attempt to address the limitations of the RASP and the NSA, other proprioception assessment tools using a continuous scale were developed. For example, the Wrist Position Sense Test (WPST) quantifies the capacity to perceive wrist position after a passive movement [41]. The assessment device consists of a box-like apparatus with a one-degree-interval scale, a splint for the forearm and hand fixed on the base and a curtain, to avoid arm visual feedback. A pointer enables subjects to indicate perceived wrist position, without any visual feedback of wrist position. Similarly to our protocol, the aim of the WPST is to objectively quantify the position sense, in degrees, without requiring motor and cognitive abilities [41]. Experimenter biases are, however, more likely to occur. Indeed, arm movement (velocity, range) and position may vary within and between assessments while using the WPST considering that passive movement needs to be performed by the examiner. Robotic devices may offer the possibility to reduce the potential influence of experimenter on the assessment. Wrist proprioceptive function has been assessed by Cappello et al. [42] using the Wrist Robot, which is a 3-degree of freedom manipulandum. A 2-alternative forced choice paradigm was employed; participants having to verbally indicate which of two different displacements was larger. As stimuli were presented consecutively, working memory may however be a confounder in this assessment. A 2-alternative forced choice paradigm was also used to evaluate the ability to detect arm displacement controlled by a planar robot [33]. This protocol also solicited memory to recall stimuli for comparison. Memory deficits would not interfere in the matching task developed by Rinderknecht et al. [43]. After a passive movement performed by a robotic device, participants had to indicate the perceived wrist position by adjusting the gauge indicator position on a touchscreen. Ingemanson et al. [44] demonstrated that finger proprioception assessment might also be possible through the use of an exoskeleton. In this task, participants were instructed to press the spacebar on a keyboard when they perceived their index and middle fingers were overlapped during a passive crisscross movement. This task assesses “dynamic position sense” at the fingers, but is neither finger- nor joint-specific. Dukelow et al. [25] quantified upper limb position sense of healthy and stroke individuals using a robotic arm position-matching task. A robot moved participants’ arm to one of the nine positions and they had to mirror-match that position with the opposite upper limb. In this matching-task, proprioceptive deficits in the contralateral upper limb could however bias the interpretation of results. In all these robotic-based proprioception assessment tasks, the involvement of the examiner was minimal.

Our assessment protocol took into consideration some of the potential confounders, since it enables the quantification of elbow position sense without using the opposite arm, involving active movement of the evaluated limb or relying on working memory. Another bias may however be introduced with the proposed approach. Since the identification of elbow relative position involves a comparison with a virtual representation, it involves a judgement based on proprioceptive and visual information. Multimodal sensory integration processes can be impaired in populations with central nervous system lesion, such as stroke.

### Position sense thresholds in healthy population

A few studies had quantified proprioception in healthy populations. It is surprising to note that position discrimination or detection thresholds obtained in other studies are in the same order of magnitude as the results obtained in the present study (7.0° ± 2.4° and 5.9° ± 2.1° for Sessions 1 and 2, respectively), despite substantial differences in experimental protocol and evaluated joint. For the upper extremity, Adamo et al. reported absolute errors ranging from 2.2° ± 1.6 to 6.0° ± 2.5°, depending on the amplitude of the movement, when elbow joint position sense was tested with a contralateral concurrent matching task [45]. Carey et al. focused on wrist position identification following a passive movement administered by the examiner [41]. The mean difference between the real position and the estimated position (20 trials) was 6.1° ± 1.8° in healthy individuals. A comparable absolute error of 5.9° ± 3.1° was observed with a similar protocol, but this time a robotic apparatus passively moved the upper limb [43]. In another study of wrist proprioception, an absolute error of 4.7° ± 0.3° was measured when participants were asked to actively reproduce the wrist configuration previously reached after a movement executed by a robotic device [46]. For the lower extremity, Venancio et al. evaluated knee position sense by looking at the angular differences between an initial reference position and the position actively reproduced by healthy participants [47]. After three trials, a mean relative error of 5.8° ± 4.4° was obtained while targeting angular positions located between 40° and 60° of knee flexion.

A cut-off of 75% in the assessment protocol was used to determine the discrimination threshold. This cut-off was previously used in other studies [32, 33]. This choice may have influenced the magnitude of the discrimination threshold. A cut-off corresponding to a lower percentage of successful trials would likely result in a lower threshold. As an example, a different percentage was used in a study aiming at the identification of ankle movement detection thresholds [31]. Indeed, the authors justified their decision of using a 50% cut-off with their protocol design, which was a voluntary choice design, instead of a two-alternative forced choice. They obtained a movement error detection threshold of 5.3° ± 2.1°.

### Test-retest reliability

In the present study, a difference of less than 2° was obtained between both assessments. Even if the magnitude of this difference seems quite small, it was statistically significant. Three sources of bias may explain the difference between thresholds measured in Sessions 1 and 2: the evaluator, the assessment tool and the participant. Different strategies were adopted to minimise potential biases induced by these factors. First of all, the assessment protocol involved a minimal contribution of the evaluator, which was the same in both assessments. All evaluators were trained to use the protocol, the robotic device, as well as the analysis softwares. Regarding the assessment protocol, the position adjustment parameters used in the first assessment were reproduced in the second. Before each assessment, the participant’s arm was calibrated following the BKIN Technologies’ recommendations. The same decision tree was used during both assessments. Finally, the difference between threshold measured in Sessions 1 and 2 may be explained by real change in participants’ capacity to discriminate their elbow position. Results of the Bland-Altman analysis showed a negative shift in the difference scores related to the mean threshold. This significant bias may reflect the presence of a learning effect. A similar effect was observed in a study investigating test-retest reliability of several KINARM robot tasks characterizing sensorimotor and/or cognitive performance [48]. In this study, an increased performance in some parameters was observed between the first and the second sessions, but not between the second and the third session. In an attempt to limit the anticipated learning effect, the present protocol included a familiarization phase; it might not have been enough, however. Implementing a practice session before testing may eliminate this learning effect. Future studies need to include more practice trials or an additional session to limit the learning effect.

Test-retest mean difference obtained in the present study (1.1°) was smaller than the error highlighted when using other common clinician tools measuring range of motion in degrees. For example, test-retest reliability of a standard manual goniometer was investigated in individuals who undergone surgery after elbow, forearm or wrist injury. The mean difference between both assessments was 3.2° for elbow flexion, and 3.5° for elbow extension [49].

The intraclass correlation coefficient calculated for our proprioception assessment protocol reflected a fair to good relative reliability. This result is similar to the test-retest reliability of the most frequently used standardised sensory assessments with stroke population [16]. The NSA presents a fair to good intra-rater agreement for the elbow’s position sense task in patients who sustained a stroke, as suggested with a Kappa coefficient of 0.71 [50]. For the RASP, Pearson’s correlation coefficients were 0.83 and 0.50 for the proprioceptive movement discrimination and for the direction discrimination, respectively. The WPST was characterized by an excellent test-retest reliability (*r* = 0.88–0.92) when administered to a population of stroke survivors [41]. Test-retest reliability was also investigated in a few robot-based assessment protocols. In Dukelow et al. [25], a moderate to excellent inter-rater reliability (*r* = 0.70–0.86) was obtained when participants with a stroke underwent 2 robotic assessment sessions, within a few minutes, conducted by two different examiners. Rinderknecht et al. [43] reported an ICC of 0.68, corresponding to a fair to good reliability, for the absolute error obtained with a robotic assessment of wrist proprioception in healthy subjects.

While a better reliability score would have been appreciated, a moderate ICC was expected, considering the relative homogeneity of the sample consisting exclusively of healthy young adults. Indeed, low levels of between-subjects variability in discrimination thresholds had an impact on ICC calculation [37]. The ICC is therefore likely to be higher when calculated with data collected in a population with proprioceptive deficits.

While the ICC is an estimate of relative reliability, the SEm provides an indication of absolute reliability. The latter quantifies the precision of individual score on a given assessment with the same units as the measurement [37]. A SEm of 1.5° was obtained in the present study, which represents 25.4% of the mean discrimination threshold measured in the second session. A SEm of 2.7°, which represents 44.3% of the mean absolute error (6.1°), was obtained with the WPST [41]. Rinderknecht et al. [43] have reported a SEm of 2.1°, corresponding to 35.6% of the mean absolute error (5.9°). The protocol proposed in the present study seems, therefore, to offer a more accurate measurement in degrees than these two protocols.

### Limitations of the study and future directions

Our position sense assessment protocol enables the quantification of elbow position sense without using the opposite arm, involving active movement of the evaluated limb or relying on working memory, considering sensorimotor and cognitive functions may be impaired after a stroke. Other consequences of stroke, such as visual disorders, extreme fatigue, hemispatial neglect, and attentional deficits, may render the use of the proposed assessment protocol difficult, even impossible. Each approach has its own limitation, and therefore it is likely that different approaches could bring a complementary perspective on proprioceptive deficits after a stroke. Taking into account that most functional activities involve multiple joint movements in three dimensions, the fact that this assessment protocol evaluated a single upper limb joint in the horizontal plane should be considered as a limitation. In addition, the possibility that some participants contracted their muscles during the experiment cannot be eliminated, despite clear instructions asking them to maintain their arm as relaxed as possible and to avoid helping the exoskeleton to execute the movement. Upper limb muscle activation should have been monitored with electromyography surface electrodes to make sure that participants remain passive.

Another limitation is that this assessment protocol needs specialized and expensive equipment, physical space, time to administer and expertise to collect and analyse the data, which could limit its clinical application. Testing duration of this proprioception assessment (approximately 40–45 min) is too long to be considered for clinical purposes. Other quantitative proprioception tools (robotic or non-robotic) have a significantly shorter assessment time, ranging from 2.5 min to 15 min [41, 43, 51–53], thus demonstrating their potential for application in clinical settings. Nonetheless, there are ways to potentially reduce the duration of the proposed assessment protocol without deteriorating its reliability. For example, increasing velocity of angular displacement could contribute in reducing assessment duration. In the proposed assessment, a velocity of 5 deg./s was used to move the arm between initial and target position. An increased velocity could be tested in further studies, while keeping in mind that inducing adverse effects such as spasticity must be avoided while assessing persons with central nervous system lesion. Moreover, a different adaptive psychophysical method, such as the Parameter estimation by Sequential Testing (PEST; [28]), may increase the precision of the threshold while minimizing the number of trials required to estimate it and consequently reducing assessment duration. The proposed robotic-based protocol may currently find utility as a research tool for future studies focusing on the effectiveness of innovative rehabilitation approaches on proprioception in different populations who sustained a central nervous system lesion. Since the sample size was limited and the data were collected specifically in healthy young adults, results of the present study cannot be generalized to older adults or individuals who had a stroke. Future research is therefore needed to validate this assessment protocol in these populations.

## Conclusion

Considering that there is a need for proprioception assessment tool adapted for persons presenting multiple deficits following a stroke, we develop a new protocol enabling the assessment of position sense without using the opposite arm and without relying on working memory. As a preliminary step, it was demonstrated that this assessment protocol can objectively quantify elbow position sense (in degrees) of healthy young adults, with a fair to good test-retest reliability. After some modifications, such as including a longer familiarization phase and the addition of muscle activation monitoring, this elbow position sense assessment protocol will be tested in older adults and individuals who sustained a stroke.

## Abbreviations

95% CI: 95% confidence interval
ICC: Intraclass Correlation Coefficient
KINARM: Kinesiological Instrument for Normal and Altered Reaching Movements
NSA: Nottingham Sensory Assessment
RASP: Rivermead Assessment of Somatosensory Performance
SD: Standard Deviation
SEm: Standard Error of Measurement
VR: Virtual reality
WPST: Wrist Position Sense Test
